# Fascial Plane Blocks Combination for Breast Surgery in Obese Patients With Difficult Airway Management: Insidious or a Valid Alternative

**DOI:** 10.7759/cureus.23652

**Published:** 2022-03-30

**Authors:** Luca Gentili, Paolo Scimia, Antonio De Cato, Franco Marinangeli, Chiara Angeletti

**Affiliations:** 1 Operative Unit of Anaesthesiology, Intensive Care and Pain Medicine, Civil Hospital G. Mazzini, Teramo, ITA; 2 Department of Clinical Medicine, Public Health and Life Science (MESVA), University of L'Aquila, L'Aquila, ITA

**Keywords:** breast surgery, obese patient, locoregional anesthesia, post operative pain management, erector spinae plane (esp) block, serratus anterior plane (sap) block

## Abstract

Obesity poses several challenges for anesthetists. The several comorbidities associated with obesity can result in very complex management, which requires a multimodal and reasoned approach. The possible difficult airways are, certainly, the obstacle that most can put the anesthetist to the test. From this point of view, regional anesthesia (RA) can be a valid alternative to general anesthesia (GA) in selected patients. The possibility of performing an anesthetic block allows the fulfilment of the surgical act. We present the case of a 56-year-old woman, with a BMI of 43. In her medical history, she has obstructive sleep apnea syndrome (OSAS) on home-oxygen therapy without continuous positive airway pressure (CPAP) therapy. The patient reported probable airway difficulties in previous breast surgery, and the preoperative evaluation highlighted and confirmed the high risk. For this reason, in agreement with the surgeons and the patient, we decided to perform RA. Forty minutes before the start of the surgery, a deep anesthetic ultrasound-guided serratus anterior plane (US-SAP; branches of the intercostal nerves in the middle axillary line [BRILMA]) was performed, followed by a right ultrasound-guided erector spinae plane (US-ESP) block. Mild sedation with propofol 1 mg/kg/h was administered and SpO_2_ always remained above 97% with nasal oxygen at 3 l/min. The surgery was completed in 35 minutes, the patient complained of no pain, and received opioid rescue therapy during the post-operative period. This case presents clinical evidence that RA can help in avoiding some dreadful complications that can occur during GA in obese patients. In any case, the anesthetic management choice must be carefully reasoned, considering the patient's clinical conditions, surgical needs, and, not least, the skills of the anesthetist.

## Introduction

Obesity represents one of the most important public health diseases of our time and is described as an abnormal/excessive accumulation of fat that causes various diseases. The anesthetic management of obese patients poses various problems. First is the choice of the technique to be used, which involves the least risk for the patient.

Regional anesthesia (RA) can represent a valid alternative to the difficulties that could be encountered with the execution of general anesthesia (GA) in selected surgical settings, such as expected difficult intubation (impaired by a lack of training and experience in surgical airway procedures [1]), cardiovascular depression, nausea, vomiting, and postoperative pain control [2]. RA techniques are attractive options for these patients with specific anesthetic problems, reducing possible risks. The main RA advantages are represented by the loss of sensitivity without compromising consciousness and the central control of vital functions. Recently, the American Society of Anesthesiologists (ASA) published practical guidelines that encourage the choice of peripheral nerve blocks (PNB) when appropriate for patients with obesity-related complications [3].

Obesity had specific challenges for the RA techniques, such as the need for special equipment, placement, and palpation of anatomical landmarks made more difficult, an increased rate of block failure (1.6 times more likely [4]), and the occurrence of side effects and complications. If RA becomes inadequate during surgery, the need for GA induction and securing an airway may be more problematic than in ideal conditions.

Ultrasound (US) can help anesthesiologists by facilitating the procedures, increasing the success rates, decreasing the rates of intra-procedural trauma and the percentages of side effects/complications thanks to the possibility of direct visualization of the characteristics of the anatomical structure and the needle with its orientation in obese patients as well as in other patients [5]. It is undeniable how the routine introduction of ultrasound has revolutionized RA and how it has been fundamental in the development of fascial plane blocks, in which a local anesthetic (LA) is injected into a tissue plane rather than directly near the perinervous space. The blocks of the thoracic wall have been the ones that have benefited most from the use of ultrasound, leading to the introduction of new techniques such as the pectoralis nerve (PECS I - II) blocks, the serratus anterior plane (SAP) block, and the erector spinae plane (ESP) block, widely used in breast surgery [6].

The patient provided written consent for data collection and publication of this report.

## Case presentation

We reported a case of the 56-year-old woman with a height of 160 cm, a weight of 110 kg, and a BMI of 43 kg/m^2^, scheduled for a right sentinel lymph node biopsy. The patient, in addition to obesity, presented a history of quadrantectomy plus radiation therapy (QUART), hypokinetic syndrome secondary to bone metastases, diabetes mellitus treated with insulin, hypertension on olmesartan, lower limb edema, mild obstructive sleep apnea syndrome (OSAS), and home-oxygen therapy (3 L/min). No CPAP therapy is required at home. The preoperative evaluation highlighted the probable difficulty in managing the airways with macroglossia, a Mallampati score of 4, El-Ganzouri Risk Index (EGRI) = 9, neck circumference >35 cm, and reported difficulty in previous breast surgery.

**Figure 1:**
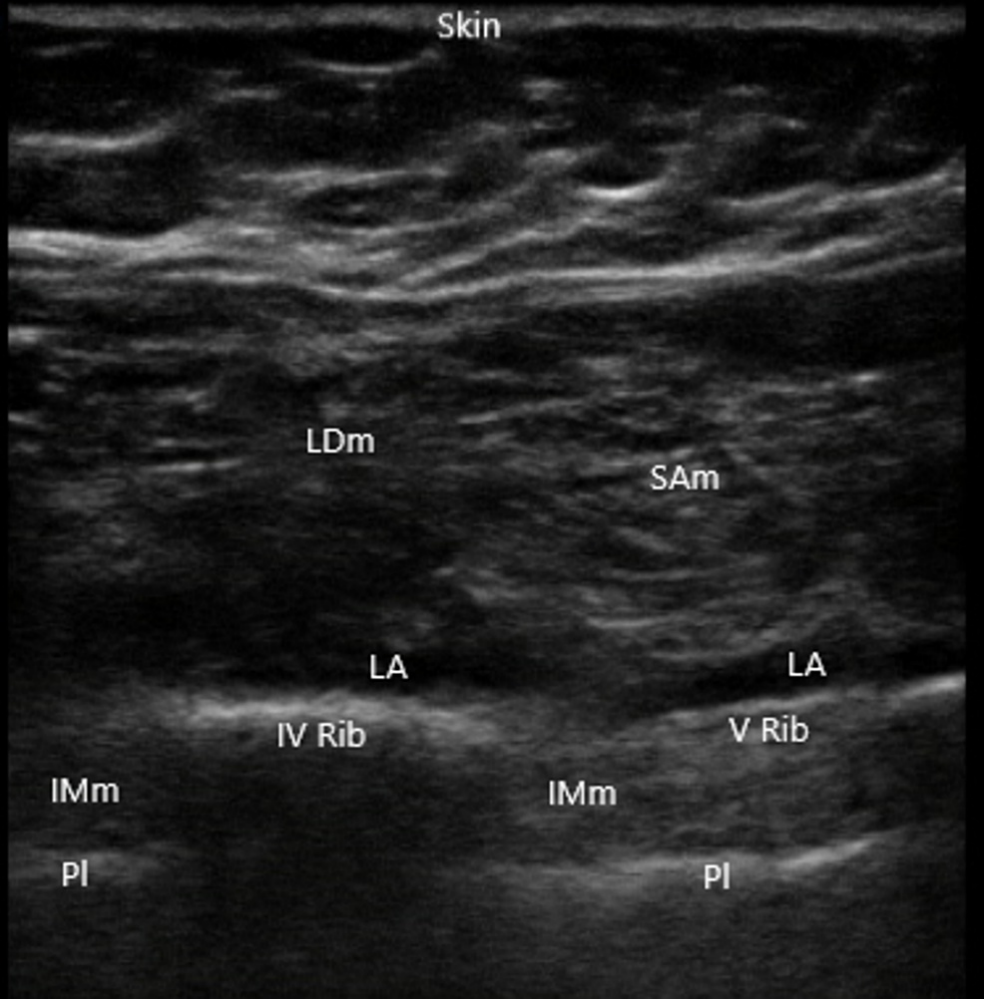
Serratus anterior plane-block A linear probe 10-12 MHz was placed in a longitudinal scan between IV and V ribs over the right mid-axillary line to visualize ribs, LDm, SAm, and IIm muscles. Via in-plane approach 30 mL of levobupivacaine 0.5% are injected in a caudo-cranial direction. LDm: latissimus dorsi muscle; SAm: serratus anterior muscle; IMm: intercostal muscles; LA: local anaesthetic; Pl: pleura.

Forty minutes before the start of the surgery, the patient received standard ASA monitors (heart rate, non-invasive blood pressure, oxygen saturation, ECG, and temperature) and was placed in the lateral position with the site of surgical interest uppermost. The patient received light sedation with midazolam 1 mg intravenous (IV) and fentanyl IV 100 mcg, and a deep SAP block for blocking the branches of the intercostal nerves in the middle axillary line (BRILMA) was performed first [7]. A linear probe, 10-12 MHz (SonoSite Edge II Ultrasound System, Fujifilm, Tokyo, Japan), was placed in a longitudinal scan between IV and V ribs over the right mid-axillary line to visualize ribs, latissimus dorsi (LDm), serratus anterior (SAm), and intercostal (IIm) muscles (Figure 1).

**Figure 2:**
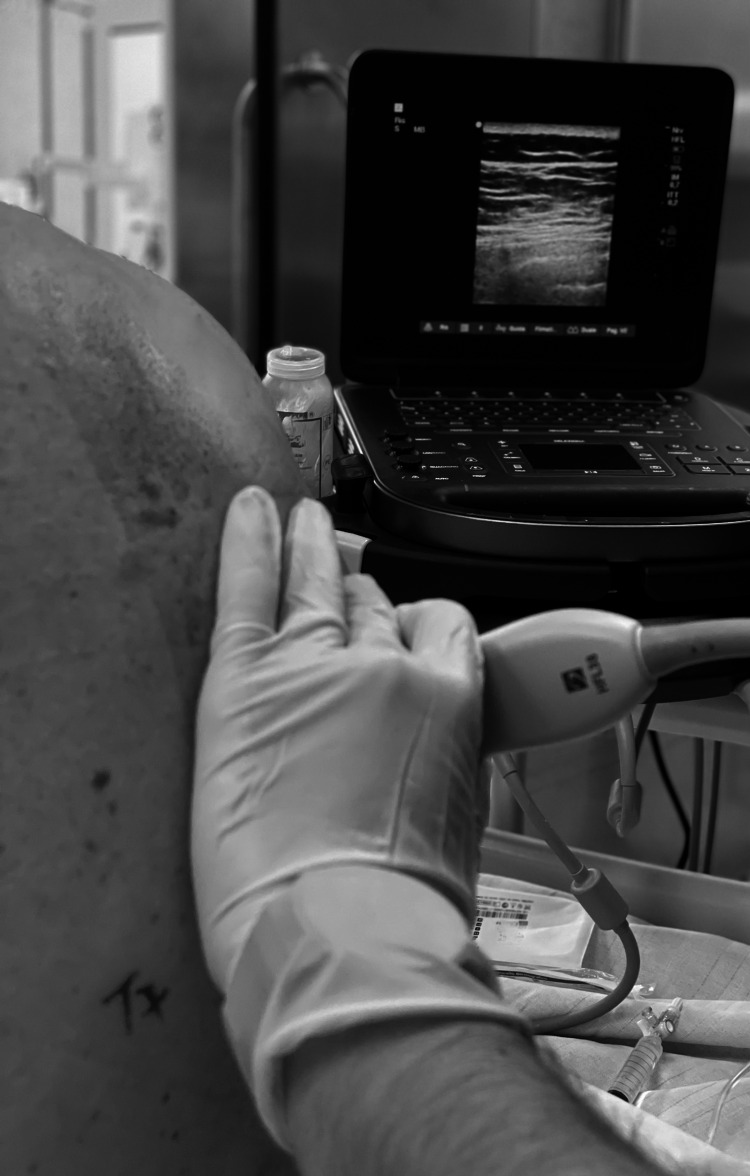
Erector spinae plane-block: patient and probe's position The probe was placed over the anatomical landmark of T7 (inferior angle of the scapula), scanning the transverse process of T6. T7: anatomical landmark of T7.

A dose of 150 mg of levobupivacaine 0.5% in 30 ml of saline solution was injected in the fascial plane between the serratus anterior and intercostal muscles, via an in-plane caudo-cranial approach to promote the spread of LA towards the axillary cavity. Later, the patient was placed in a sitting position, a high-frequency linear ultrasound transducer was placed in a longitudinal orientation 3 cm lateral to the T6 spinous process, and a right ESP block was performed [8] (Figure 2).

**Figure 3:**
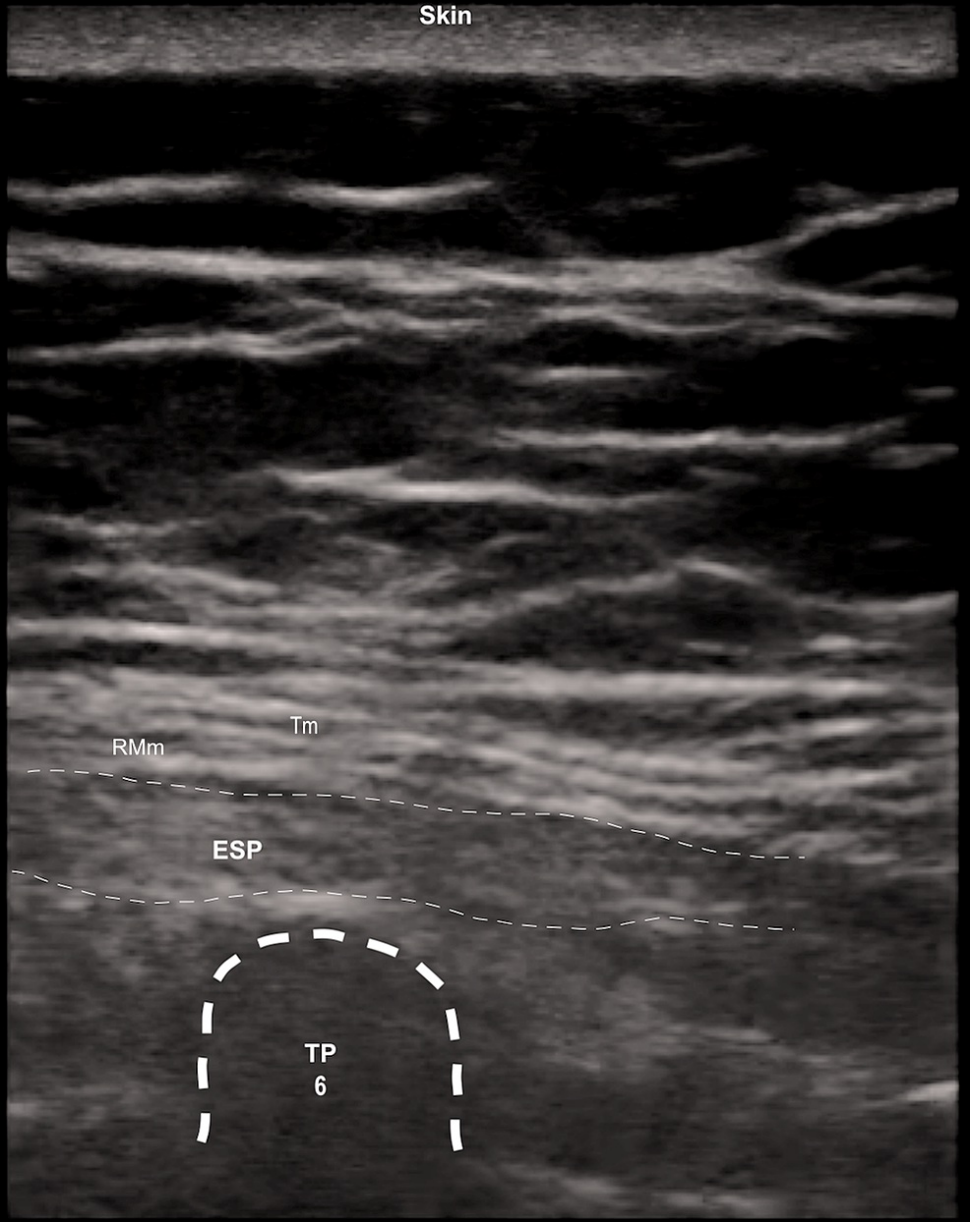
Erector spinae plane-block A linear probe 10-12 MHz was placed in a longitudinal orientation 3 cm lateral to the T6 spinous process. Three muscles were identified superficial to the hyperechoic transverse process shadow as follows: Tm, RMm, and ESP. Via in-plane approach 20 mL of levobupivacaine 0.25% are injected in a caudo-cranial direction. Tm: trapezius muscle; RMm: rhomboid major muscles; ESP: erector spinae muscle; TP 6: transverse process of T6.

Three muscles were identified superficial to the hyperechoic transverse process shadow as follows: trapezius (Tm), rhomboid major (RMm), and erector spinae (ESP). A 9-cm 21-gauge block needle (Temena UPC 90-22G-Ambu - Regional Anesthesia) was inserted until the tip touched the transverse process T6. Then 50 mg of levobupivacaine 0.25% in 20 mL of saline solution were injected via an in-plane caudo-cranial approach, confirming the linear spread of LA between the anterior layer of the erector spinae muscle and the tip of the transverse process (TP) T6 (Figure 3).

Fascial blocks were performed by a trainee specialist under supervision. Propofol at 1% was intravenously administered for sedation, with the Bispectral Index System (BISTM) between 75 and 90, at a dosage of 1 mg/kg/h. Nasal oxygen was administered via an end-tidal CO_2_ nasal cannula (Microsteam - Smart CapnoLine® H Plus O_2_) at 3 L/min. EtCO_2_ ranged from 36 to 45 mmHg and SpO_2_ always remained above 97%. After 15 minutes, sensory anesthesia from T2 to T8 was observed by a pin-prick test with a hypodermic needle. Although the pin-prick test confirmed the sensory block, we were prepared for the possibility of having to deepen the anesthetic plan or even having to resort to GA. We, therefore, worked on possible management of the anticipated difficult airways. We provided all the necessary devices (laryngeal mask, Frova introducer, videolaryngoscope, cricothyrotomy set), including the possibility of awake intubation. In fact, we made the fibrobronchoscope available and alerted an expert operator, according to the most recent guidelines. In addition, surgeons were warned about eventual surgical airway management [1,9,10].

The surgery was completed in 35 minutes, the patient complained of no pain, the hemodynamic parameters remained stable, and no opioids were administered. In the post-operative period, after 3- and 12-hour, reevaluation of pain was recorded with NRS=0/2 both in static and dynamic settings, only a supplementary dose of paracetamol 500 mg was administered. The same patient, in the postoperative period, expressed satisfaction with the efficacy and comfort deriving from the anesthetic technique chosen.

## Discussion

The anatomical and physiological changes associated with obesity require careful and accurate anesthetic and surgical evaluation. In fact, changes in the body structure caused by obesity can reduce accessibility in several anatomical sites, increasing the complexity of the maneuvers in the perioperative management, resulting in an absolute increase of complications affecting the airways, gastrointestinal, neurological, cardiovascular, and respiratory systems.

For the anesthetist, management of the airways represents one of the crucial and fundamental moments for patients to be subjected to anesthesia, especially in the case of obese patients. Obesity is even more associated with a series of factors that can affect both ventilation and endotracheal tube placement. Factors linked to obesity that can lead to the management of a difficult airway are represented by patient position, presence of macroglossia, volumetric increase in retropharyngeal tissues, anatomical changes in the neck region (wide and short neck), reduction of thyromental and thyrochest wall distance, and cervical or jaw mobility. In such cases, the correct approach, as suggested by the major guidelines, was represented by awake intubation with fibrobronchoscopy, a proven and safe method for predicting difficult airways (as previously organized in perioperative planning). In light of these considerations, the anesthetist can opt for local and regional techniques (neuraxial or peripheral nerve block), avoiding manipulation of the airways and maintaining spontaneous ventilation [11].

RA can help in avoiding some of the dreadful complications that can occur during GA in obese patients. Nevertheless, the large percentage of RA failures in these patients compared to normal-weight subjects, which is linked to technical obstacles, must be taken into consideration. In this context, ultrasound guidance has the advantage of increasing the probability of block success in cases where the classic anatomical landmarks are difficult to see and palpate. For this reason, we decided to perform a deep SAP block instead of a superficial SAP block due to the bad sonoanatomy of the patient. In fact, the rib is clearly visible as it is hyperechoic, resulting in an easily identifiable target. Furthermore, by taking advantage of the costal surface stop of the needle tip, it limits the risk of pneumothorax and respiratory complications. A risk/benefit analysis must always guide the choice of an appropriate and individualized RA technique for the patient, taking into consideration the physical state, the comorbidities, the anatomical characteristics, the surgical interventions, and above all, the experience and technical skills of the anesthesiologist [5].

Regarding the patient described, the complex innervation of the axillary region is added to the difficulty in carrying out the locoregional anesthesia for this obese patient. In fact, the innervation of the axilla depends on the brachial plexus (C5-T1, which contributes to the pectoral, long thoracic, and thoracodorsal nerves) and the lateral cutaneous branches of the upper intercostal nerves (T2-T7, including the intercostal brachial nerve) [12].

Many locoregional techniques have been used to cover the axilla area, and the PECS II block and serratus plane block (SPB) are the ones that surely anesthetize the lateral branches of the intercostal nerves and long thoracic and thoracodorsal nerves [13,14]. However, we decided to perform the ESP block [8,15] to enhance the anesthetic coverage on the lateral branches of the intercostal nerves, trying to minimize the risk of failure of the SPB, although its success remains necessary for coverage of long thoracic and thoracodorsal nerves. In our case, the decision to associate ESP and SPB was taken into consideration after the preoperative summit with the surgeons who, in the event of a positive lymph node biopsy, would have opted for a large lymphadenectomy and complete emptying of the axillary cavity with a possible revision of the previous superior external quadrantectomy up to mastectomy. We were aware that the levobupivacaine concentration (0.25%) would not guarantee anesthesia but analgesia. Nevertheless, we did not want to run the risk of local anesthetic systemic toxicity (LAST) as we had reached the maximum recommended dose per patient [16]. In addition, the choice to perform an ESP block instead of a thoracic paravertebral block (tPVB) was guided by the bad sonographic window and the depth of paravertebral space of >5 cm. A recently described association of two or more techniques (ESP, SPB, PECS1-2), allowed us to perform mastectomy with sentinel lymph node biopsy with the patient only slightly sedated and without the use of opioids, neither intra nor postoperative [17-19]. Recent evidence showed that these blocks might also attenuate the surgical stress response and opioid perioperative consumption, reducing the incidence of chronic pain and the likelihood of tumor recurrence after axillary dissection [20].

The operator's lack of experience in performing paravertebral blocks has been a further reason for the combined execution of ESP and SPB for wake-up surgical anesthesia and subsequent post-operative analgesia in this patient. Both fascial blocks were performed by a trainee specialist in anesthesia under the guidance of the attending anesthesiologist. This showing as ultrasound-guided fascial blocks represents a simple procedure with analgesic efficacy and a low probability of complications that motivates use even in the hands of adequately trained and supervised specialists in training.

## Conclusions

This case report suggests that the decision to proceed with RA with or without a secure airway should be made with careful consideration of the patient and surgical and environmental factors. In our opinion, the novel US-guided interfascial thoracic blocks may be successfully used to achieve sensory block of the axilla, allowing us to avoid the risks of general anesthesia, especially in patients with expected difficulty managing airways. Nevertheless, ours is only a single case, and since the focus must always be on patient safety, this cannot be established with a single case. Further studies should be needed to confirm whether the US-guided thoracic interfascial block techniques are safe and appropriate anesthetic and analgesic alternatives for high-risk patients undergoing axillary surgery in routine clinical practice.
